# Acute orexin antagonism selectively modulates anticipatory anxiety in humans: implications for addiction and anxiety

**DOI:** 10.1038/s41398-022-02090-x

**Published:** 2022-08-02

**Authors:** Stephanie M. Gorka, Kia J. Khorrami, Charles A. Manzler, K. Luan Phan

**Affiliations:** 1 Department of Psychiatry and Behavioral Health, The Ohio State University Wexner Medical Center, 370 W. 9th Avenue, Columbus, OH 43210 USA; 2 Institute for Behavioral Medicine Research, The Ohio State University, 460 Medical Center Drive, Columbus, OH 43210 USA

**Keywords:** Biomarkers, Physiology

## Abstract

Research indicates that heightened anticipatory anxiety underlies several forms of psychopathology. Anticipatory anxiety can be reliably and objectively measured in the laboratory using the No-Predictable-Unpredictable (NPU) threat paradigm. The NPU paradigm is an ideal research tool for the NIH ‘Fast-Fail’ approach of screening promising compounds and testing human target engagement. Evidence from preclinical studies suggests that the hypocretin/orexin (ORX) hypothalamic neuropeptide system is a potential means for modulating anticipatory anxiety and disrupting stress-related alcohol use. The current study tested this question using a psychophysiological probe of the ORX system in humans. We examined whether a single dose of suvorexant (SUV; 10 mg; dual ORX receptor antagonist) can effectively and selectively target a well-validated human laboratory index of exaggerated anticipatory anxiety using a within-subjects placebo-controlled design. A total of twenty-one volunteers completed two laboratory sessions during acute administration of 10 mg SUV or placebo. Across sessions, we administered the NPU paradigm probing sustained anticipatory anxiety and fear while startle eyeblink was recorded as an index of aversive reactivity. Questionnaires assessing mood states and subjective drug effects were also collected. Results indicated SUV was well-tolerated. Compared with placebo, SUV was associated with decreased startle reactivity during anticipatory anxiety but not fear or no-threat conditions. Therefore, SUV selectively and effectively reduced objective indicators of anticipatory anxiety in humans and engaged our laboratory target of psychopathology. ORX antagonism may be a promising strategy for modulating human anxiety and potentially, stress-related alcohol use.

## Introduction

Converging research indicates that heightened reactivity to uncertain threat (U-threat) underlies several forms of psychopathology. U-threat is typically defined as a threat that is unpredictable in its temporality, intensity, frequency, or duration. U-threat elicits a generalized feeling of apprehension and hypervigilance that is not associated with an identifiable source, referred to as anticipatory anxiety [1, 2]. U-threat is in contrast with predictable threat (P-threat), which is signaled by a discrete cue and elicits a phasic response to an identifiable stimulus that is time-locked to the threat [1, 2]. U-threat and P-threat produce distinguishable aversive states [3] that are mediated by overlapping, but separable, neural circuits [4, 5]. Fear and anxiety, and U-threat and P-threat, are related though accumulating evidence points to the fact that individual differences in reactivity to U-threat (but not P-threat) play a role in the onset and maintenance of multiple forms of psychopathology, particularly fear-based anxiety disorders (e.g., panic disorder, social anxiety disorder, phobias) [6] and addiction [7].

In order to induce anticipatory anxiety and fear in the laboratory, Grillon and colleagues developed the No-Predictable-Unpredictable (NPU) threat task [8]. The NPU is a translational paradigm with three within-subjects conditions: no-shock (N), predictable electric shock (P), and unpredictable electric shock (U). The task includes bursts of white noise used to elicit and record the startle eyeblink reflex, a highly reliable and easy-to-record index of aversive reactivity [9, 10]. Using an adapted version of the NPU task, our lab has repeatedly demonstrated that fear-based anxiety disorders and alcohol use disorder (AUD) are characterized by exaggerated startle eyeblink potentiation to U-threat, but not P-threat, compared with matched and healthy controls [6, 7, 11]. Startle magnitude during U-threat correlates with severity of anxiety and AUD symptoms and stress-related coping motives for alcohol use [6, 7]. Meanwhile, alcohol intoxication selectively and effectively dampens reactivity to U-threat [12, 13].

We have also shown the NPU paradigm is sensitive, reliable, and reflective of frontolimbic brain function [14, 15]. Consequently, the NPU paradigm has been identified as an ideal research tool for mechanistic medication development [16, 17]. Recent recommendations urge researchers to use biologically-plausible, valid, and reliable human laboratory measures of pathophysiology to test promising compounds from preclinical studies to directly inform the go/no-go decision to move to a clinical trial. This strategy is intended to accelerate the pace of drug discovery and bring forth novel mechanistic pharmacotherapies. With regard to anxiety and AUD, though there are available medications to treat these disorders, they are modestly effective and do not work for everyone [18]. The development of new and more efficacious pharmacotherapies for anxiety disorders and AUD are urgently needed and a top public health priority.

To this end, one neurochemical system that has emerged as a promising target for next-generation medication development is the orexin (ORX) system. Orexins (also known as hypocretins) are peptides that are produced in the hypothalamus and regulate a range of behavioral and physiological processes including wakefulness, arousal, energy metabolism, and stress reactivity [19–21]. There are currently two known types of ORX receptors (OX1R and OX2R) that are distributed throughout the brain including networks involved in stress, fear, and anxiety [22, 23]. Preclinical studies show that ORX is involved in several key aspects of chronic anxiety including arousal and hypervigilance. For instance, ORX neurons are activated by acute threat, and infusion of orexin directly into the brain induces anxiety-like behaviors [24, 25]. Meanwhile, decreases in ORX signaling blunts stress and anxiety responses, particularly during challenges that elicit arousal [26–28]. In humans, individuals with anxiety disorders have higher serum and cerebrospinal fluid ORX levels compared with controls [28, 29]. A recent study in those with panic disorder reported a gene variant linked to ORX system functioning (*HCRTR1)* is assocaited with increased avoidance and arousal-based anxiety symptoms [30]. In sum, evidence clearly indicates ORX plays a critical role in arousal-based anxiety; though there are only a few studies in humans testing these theories.

Accumulating preclinical data also indicates the ORX system is involved in alcohol abuse. Antagonism of ORX receptors has been repeatedly shown to decrease alcohol self-administration and relapse-like behavior [31, 32]. For example, administration of SB334867 (SB) (ORX1R antagonist) was found to decrease alcohol consumption, block alcohol condition place preference, and prevent cue-induced reinstatement of alcohol seeking [33, 34]. In mouse models, ORX antagonism similarly decreases binge-like alcohol consumption and escalated alcohol intake due to cycles of chronic intermittent ethanol exposure [35, 36]. In many studies, the effect of OXR antagonism was most robust in animals exhibiting high motivation for alcohol, such as those genetically selected for high alcohol preference [37] or rats with natural high propensity for alcohol [38]. Given that high motivation for alcohol is characteristic of human AUD, these data are promising for human translation. There are also studies showing that SB treatment is particularly effective at reducing stress-induced alcohol relapse-like behavior [39], and that ORX is involved in the emotional dysregulation that occurs during alcohol withdrawal [40]. These data are consistent with the role of ORX in regulating both arousal and alcohol behavior and the interplay between anxiety and AUD symptoms.

ORX antagonism may be an effective strategy for reducing chronic hyper-arousal and disrupting the negative reinforcement cycle of AUD and anxiety; though no study has directly addressed this question. To date, the dual ORX receptor antagonist suvorexant (Belsomra™), used to treat insomnia, is an FDA-approved ORX receptor antagonist. Consistent with the NIH ‘Fast-Fail’ mission, the present study was a small, proof-of-concept psychophysiological probe of the ORX system in humans. We examined whether a single dose of suvorexant (SUV; 10 mg) can effectively and selectively target a well-validated human laboratory index of exaggerated anticipatory anxiety using a within-subjects placebo-controlled design. A dose of 10 mg was selected because it is the lowest effective clinical dose, has negligible cognitive and emotional side effects, and has not been associated with adverse events [41]. If ORX antagonism selectively engages our U-threat laboratory target, without modifying reactivity to P-threat, there would be compelling human evidence that ORX is a neurochemical modulator of anticipatory anxiety, and ORX antagonism would be a promising pharmacological target for anxiety disorder and/or AUD medication development.

## Method

### Participants

Volunteers were recruited from the community via flyers and online advertisements for an open-label pharmacological challenge laboratory study. To be included in the study, individuals were required to be between ages 18 and 30 years, generally medically healthy, and able to provide written informed consent. Exclusion criteria included: (a) clinically significant medical or neurologic condition, (b) active suicidal ideation, (c) current or recent (past 2 months) use of any psychoactive medications or antihistamines, (d) current use of strong CYP3A live enzymes or moderate CYP3A inhibitors or strong CYP3A inducers, (e) current use of digoxin, (f) currently pregnant, not using contraception, nursing, or trying to become pregnant, (g) obesity as defined by a body-mass index equal or greater than 30, (h) current substance use disorder, (i) current or past major depressive disorder, psychotic disorder, bipolar disorder, or obsessive-compulsive disorder, (j) deafness in either ear, (k) moderate to severe traumatic brain injury, (l) smoke 5 or more cigarettes (or electronic equivalent) per day and susceptible to acute nicotine withdrawal, (m) positive breath alcohol screen the lab of the lab visits, and (n) unwillingness to abstain from driving or engaging in any activities requiring full alertness within 24 hours of lab visits. All participants provided written informed consent and the protocol was approved by the university Institutional Review Board. Participants were monetarily compensated for their time.

A total of 21 participants (9 males, 12 females) enrolled in the study. Participants were 21.6 (±2.2) years old. Ethnic and racial composition of the sample included the following: 4.8% Hispanic, 71.4% Caucasian, 23.8% Asian, and 4.8% ‘biracial’. No participant met diagnostic criteria for any current mood, anxiety, or substance use disorder. All participants completed both laboratory sessions and had good quality startle eyeblink data (i.e., at least 4/10 good blinks in each task condition).

### Drug and placebo

Capsules were obtained from a compound pharmacy and dispensed by the university Investigational Drug Service (IDS). All capsules were opaque and identical in appearance. Drug capsules included 10 mg of SUV (i.e., Belsomra®; Merck & Co., Inc.) with dextrose filler. Placebo capsules included only dextrose filler. Capsules were administered to subjects in double-blind conditions under medical supervision. Drug order was randomized by the university IDS prior to data collection. A total of 11 subjects received SUV at their first session and 10 received placebo. Randomization was therefore successful.

### Procedure

Each participant completed three study visits including a video-conference screen and two in-person lab visits. Study visits were 2–7 days apart. The screen included informed consent, a short battery of questionnaires, and the Mini International Neuropsychiatric Interview (MINI).

Each lab visit began at 8:30 am. Participants were instructed to abstain from food and beverage other than water 2-hours prior to the session. Upon arrival, participants provided a negative breath and urine screen for alcohol, pregnancy (women), and recent illicit drug use. The placebo or drug capsule was administered while nursing staff monitored vital signs every 30-mins. Peak plasma concentration for SUV is 2 hours post-ingestion. All psychophysiological laboratory assessments were administered during peak concentration. At 240-minutes post-ingestion, participants were discharged following medical clearance from nursing staff.

### Subjective mood and drug effects

Standardized questionnaires were used to assess mood states and subjective drug effects throughout the lab sessions. Specifically, participants completed a 21-item visual analog scale (VAS) [42], Drug Effects Questionnaire (DEQ) [43], and the Profile of Mood States (POMS) [44]. The VAS ratings were made using a 0 (not at all) to 100 (extremely) scale and included the following scales of interest: anxious, tired, drowsy, and nauseous. The DEQ required participants to rate the extent to which they: (1) feel any drug effect; (2) like the drug effect; (3) feel high; and (4) would like more drug. DEQ items were rated on a 5-point Likert scale except for like drug effect which was a 9-point scale. The POMS is a 72-item adjective checklist rated on a five-point Likert Scale. The POMS yields eight subscales and two composite scales of negative arousal and positive mood. The VAS and DEQ were collected immediately before capsule ingestion and every 30-minutes afterwards. The POMS was collected immediately before capsule ingestion and every 60-minutes afterwards. At the end of each session, participants were asked to indicate which capsule they believed they received. Participants’ scores on VAS, DEQ, and POMS were averaged across each session, consistent with prior our studies [45].

### Self-reported symptoms

During the screen session participants completed the Depression, Anxiety, and Stress Scale (DASS-21) [46]. The DASS-21 is comprised of 21 items and three subscales measuring current symptoms of depression, anxiety, and stress. Respondents rate the severity of each symptom during the past week on a 0–3 scale. Participants also completed the Alcohol Use Disorders Identification Test (AUDIT) [47], which was developed by the World Health Organization (WHO) to assess hazardous and harmful alcohol use. The AUDIT includes 10-items that are combined to create a total score.

### NPU threat test

The NPU startle task and laboratory procedures have been extensively described by our group [48, 49]. In brief, shock electrodes were placed on participants’ left wrist and a shock work-up procedure was completed to identify the level of shock intensity each participant described as “highly annoying but not painful” (between 1–5 mA). Participants then completed a 2-min startle habituation task to reduce early, exaggerated startle potentiation. The task itself was modeled after Grillon and colleagues NPU threat task and thus included three within-subjects conditions: no shock (N), predictable shock (P), and unpredictable shock (U). Text at the bottom of the computer monitor informed participants of the current condition. Each condition lasted 145-s, during which a 4-s visual countdown (CD) was presented six times. The interstimulus intervals (ISIs; i.e., time between CDs) ranged from 15 to 21-s during which only the text describing the condition was on the screen. No shocks were delivered during the N condition. A shock was delivered every time the CD reached 1 during the P condition. Shocks were delivered at random during the U condition (both during the CD and ISI). Startle probes were administered during both the CD and ISI, and there was always at least 10-s between two probes or a shock and a probe. Each condition was presented two times in a randomized order (counterbalanced). Participants received 24 total electric shocks (12 in P; 12 in U) and 60 total startle probes (20 in N; 20 in P; 20 in U).

### Startle data collection and processing

Startle data were acquired using BioSemi Active Two system (BioSemi, Amsterdam, The Netherlands) and stimuli were administered using Presentation (Albany, CA). Electric shocks lasted 400-ms and acoustic startle probes were 40-ms duration, 103-dB bursts of white noise with near-instantaneous rise time presented binaurally through headphones.

Startle responses were recorded from two 4-mm Ag/AgCl electrodes placed over the orbicularis oculi muscle below the left eye. The ground electrode was located at the frontal pole (Fpz) of an electroencephalography cap that participants were wearing as part of the psychophysiological set-up. One startle electrode was placed 1-cm below the pupil and the other was placed 1-cm lateral of that electrode. Data were collected using a bandpass filter of DC-500-Hz at a sampling rate of 2000-Hz.

Blinks were processed and scored according to published guidelines: [50] applied a 28 Hz high-pass filer, rectified, and then smoothed using a 40 Hz low-pass filter. Peak amplitude was defined within 20-150-ms following the probe onset relative to baseline (i.e., average activity for the 50-ms preceding probe onset). Each peak was identified by software but examined by hand to ensure acceptability. Blinks were scored as non-responses if activity during the post-stimulus time frame did not produce a peak that is visually differentiated from baseline. Blinks were scored as missing if the baseline period was contaminated with noise, movement artifact, or if a spontaneous or voluntary blink began before minimal onset latency. Blink magnitude values (i.e., condition averages include values of 0 for non-responses) were used in all analyses.

### Data analysis plan

We first examined session differences in subjective drug effects using a series of paired samples *t*-tests. We focused on the 10 specific scales hypothesized to be affected by SUV. Next, to test our specific hypotheses, we conducted a session (2: SUV, placebo) by task condition (3: no-threat, P-threat, U-threat) repeated measures analysis of variance (ANOVA) with startle magnitude as the dependent variable. Biological sex was entered as a between-subjects covariate given that it is unknown whether SUV has differential sex effects on threat reactivity. Drug capsule order was also dummy coded and entered as a covariate to account for potential treatment order effects.

An a priori power analysis was conducted using G*Power version 3.1.9.7 to determine the minimum sample size required to test the study hypothesis. Results indicated the required sample size to achieve a large effect (*f* = 0.40) with 90% power, *α* = 0.05, and a conservative correlation of .40 between observations on the same subject was 20 subjects. A large effect was estimated based on prior studies demonstrating a robust modulation of startle magnitude by known anxiolytics (e.g., benzodiazepines [3]). A large effect also serves as justification for sufficient target engagement and subsequent clinical trial testing. Overall, the obtained sample size of 21 individuals was adequate to test the study hypothesis.

Post-hoc we conducted two exploratory Pearson’s correlations to examine whether SUV-related change in U-threat reactivity was associated with baseline anxiety symptoms and/or current problem alcohol use. Specifically, we correlated DASS-21 anxiety subscale scores and AUDIT total scores with percent change in startle magnitude (((placebo–SUV)/placebo) × 100))).

## Results

### Subjective drug effects

Mean subjective ratings for each scale, averaged across each session, are presented in Table 1. Administration of SUV was associated with increased reports of feeling drowsy and tired relative to placebo. Participants also reported they could ‘feel’ drug effects to a greater extent during administration of SUV compared with placebo. SUV was associated with reductions in negative arousal and positive mood relative to placebo. Overall, administration of SUV was well-tolerated with no adverse events including excessive drowsiness. With regard to blinding, 61.9% successfully reported receiving drug and 85.7% successfully reported receiving placebo.

**Table 1:** Subjective reports during each session.

	PBO	SUV	Comparison
DEQ Feel	0.2 (0.3)	0.9 (0.5)	t (20) = 5.73, *p* < 0.01
DEQ Like	<0.0 (0.1)	<0.0 (0.7)	t (20) = −0.20, *p* = 0.85
DEQ High	<0.0 (0.1)	0.2 (0.4)	t (20) = 2.08, *p* = 0.05
DEQ Want	1.5 (0.7)	1.5 (0.8)	t (20) = 0.55, *p* = 0.59
VAS Anxious	10.3 (9.5)	13.2 (12.1)	t (20) = 2.03, *p* = 0.06
VAS Tired	26.9 (22.6)	45.8 (22.1)	t (20) = 4.29, *p* < 0.01
VAS Drowsy	19.7 (19.7)	40.0 (25.0)	t (20) = 4.18, *p* < 0.01
VAS Nauseous	3.9 (6.4)	6.3 (10.4)	t (20) = 1.76, *p* = 0.09
POMS Negative Arousal	9.0 (4.6)	3.0 (5.6)	t (20) = −4.70, *p* < 0.01
POMS Positive Mood	7.7 (4.1)	6.3 (2.7)	t (20) = −2.32, *p* = 0.03

*DEQ* Drug Effects Questionnaire, *VAS* Visual Analog Scale, *POMS* Profile of Mood States.

### Startle effects

**Fig. 1:**
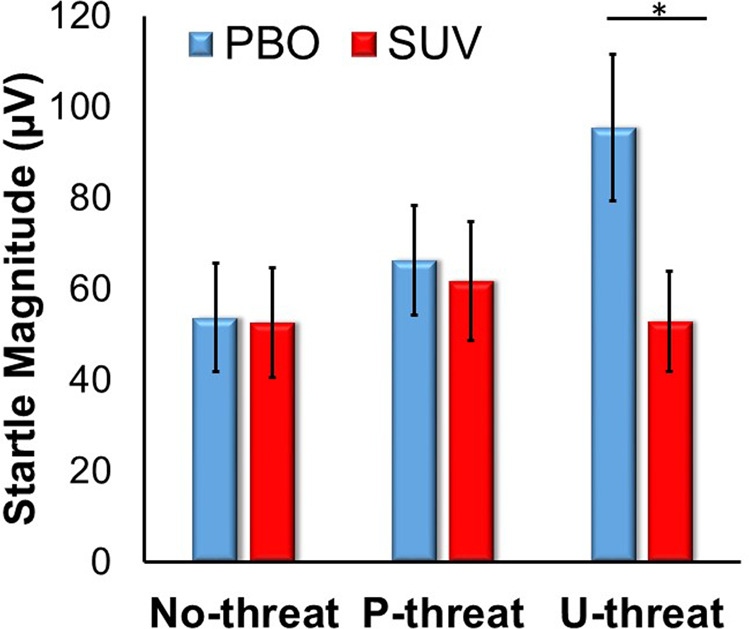
Bar graph displaying mean startle magnitude during each task condition, across both lab sessions. PBO placebo, SUV 10 mg suvorexant. Bars reflect two standard errors.

Results of the omnibus repeated measures ANOVA are presented in Table 2. There was a main effect of session such that startle was lower during SUV relative to placebo. There was also the expected main effect of task condition. Startle during U-threat (*F*[1,17] = 9.15, *p* = 0.008, *η*_G_^2^ = 0.350) and P-threat (*F*[1,17] = 6.03, *p* = 0.025, *η*_G_^2^ = 0.262) was greater than startle during No-threat. In addition, startle during U-threat was greater than startle during P-threat (*F*[1,17] = 5.17, *p* = 0.036, *η*_G_^2^ = 0.233). These main effects were qualified by a significant session by task condition interaction (Fig. 1). Startle was lower during SUV relative to placebo during U-threat (*F*[1,17] = 13.12, *p* = 0.002, *η*_G_^2^ = 0.435). However, there was no effect of session on startle during P-threat (*F*[1,17] = 0.61, *p* = 0.446, *η*_G_^2^ = 0.035) or No-threat (*F*[1,17] = 0.10, *p* = 0.761, *η*_G_^2^ = 0.006).

**Table 2:** Results of omnibus repeated measures ANOVA.

	Sum of squares	df	Mean square	*F*	*p* value	Partial Eta squared
Sex	5952.7	1, 17	5952.7	0.28	0.605	0.016
Drug order	17310.9	1, 17	17310.9	0.81	0.381	0.045
Session	6330.5	1, 17	6330.5	5.99	0.026	0.260
Session × sex	1069.7	1, 17	1069.7	1.01	0.329	0.056
Session × drug order	12.2	1, 17	12.2	0.01	0.916	0.001
Sex x drug order	386.9	1, 17	386.9	0.04	0.851	0.002
Session × sex × drug order	314.8	1, 17	314.8	0.30	0.592	0.017
Task condition*	7020.5	2, 34	3510.3	7.56	0.002	0.308
Task condition × sex	238.1	2, 34	119.1	0.26	0.775	0.015
Task condition × drug order	646.6	2, 34	323.3	0.70	0.505	0.039
Task condition × sex × drug order	98.0	2, 34	49.0	0.11	0.900	0.006
Session × task condition*	10102.2	2, 34	5051.1	11.46	<0.001	0.403
Session × task condition × sex	91.6	2, 34	45.8	0.10	0.902	0.006
Session × task condition × drug order	329.7	2, 34	164.9	0.37	0.691	0.022
Session × task condition × sex × drug order	719.8	2, 34	359.9	0.82	0.451	0.046

Session = 10 mg of suvorexant or placebo administration, task condition = no-threat, predictable threat, or unpredictable threat.

**Fig. 2:**
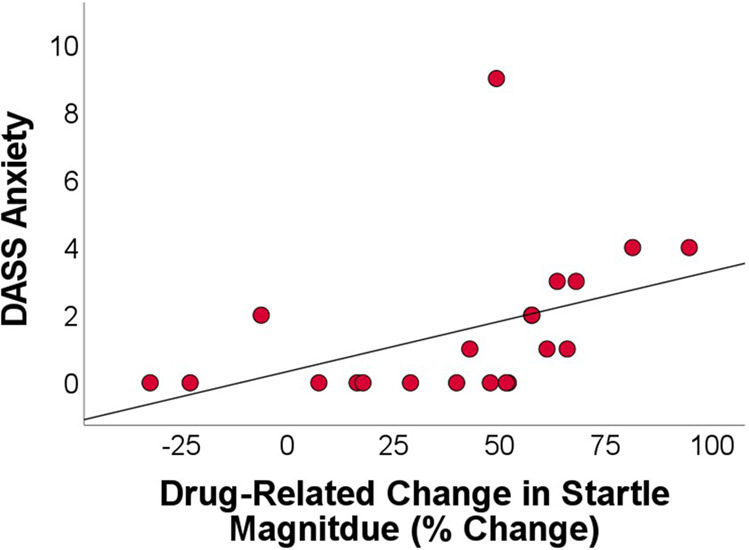
Scatter plot displaying the significant association between current anxiety symptoms and drug-related change in startle magnitude during unpredictable threat. DASS Depression, Anxiety, and Stress Scale (21 items).

On average, startle during U-threat was 40.2% (±33.0) lower during SUV compared with placebo. Greater baseline DASS-21 anxiety scores were correlated with greater SUV-related decreases in startle magnitude during U-threat (*r* = 0.44, *p* = 0.044; Fig. 2). There was no association between baseline AUDIT scores and SUV-related changes in startle magnitude (*r* = 0.16, *p* = 0.494).

We also explored whether SUV-related decreases in startle magnitude during U-threat were associated with self-reported session changes in feeling drowsy and tired.

Correlational analyses indicated there were no significant associations between startle change and tiredness (*r* = 0.19, *p* = 0.398) or drowsiness (*r* = 0.24, *p* = 0.300), further highlighting the pattern of results was not entirely driven by general sedation.

## Discussion

The current study was designed to test whether a single dose of SUV effectively and selectively modulates an objective indicator of anticipatory anxiety in humans. There is a robust animal literature suggesting the ORX system is involved in arousal-based anxiety and the motivational drive for alcohol use [51, 52]. ORX antagonism has been identified as a promising next-generation therapeutic for anxiety and AUD [53, 54]. Surprisingly, very few studies on human volunteers have directly probed the ORX system. It is therefore unclear whether prior preclinical findings translate to clinical populations and whether ORX antagonism is likely to succeed in clinical trials. In hopes of ultimately bringing forth novel mechanistic pharmacotherapies for arousal-based disorders, we aimed to implement a ‘Fast-Fail’ design using an objective laboratory target and a small unselected sample. Our results supported our hypotheses and corroborate preclinical data. Compared with placebo, a single dose of SUV decreased startle reactivity to U-threat, while sparing changes in startle reactivity to P-threat and no-threat. The magnitude of the within-subjects effect was large and specific. The study is the first to show in humans that ORX is a neurochemical modulator of objective anticipatory anxiety.

The effect of SUV administration on startle reactivity was specific to the U-threat condition. This is important given that excessive reactivity to U-threat is theorized to underlie the onset and maintenance of multiple forms of psychopathology. Pharmacotherapies that specifically dampen U-threat reactivity, without impacting other forms of threat sensitivity, are of extreme interest. Benzodiazepines dampen startle eyeblink potentiation to U-threat, but not P-threat, during the NPU paradigm and are known as effective anxiolytics [3]. Although SUV targets a different neurotransmitter system, present results suggest it has a similar selective impact on human anticipatory anxiety. ORX antagonists have limited abuse potential and a relatively safe medication profile making them attractive therapeutics [55, 56].

The current findings also revealed a significant correlation between current DASS-21 anxiety scores and SUV-related decreases in startle during U-threat. This indicates that individuals with higher levels of anxiety may gain increased benefit from SUV, or greater acute decreases in anticipatory anxiety. This relationship further highlights the potential utility of targeting the ORX system in the context of clinical anxiety. However, by design, the current sample was unselected and reported generally low levels of anxiety. The correlational finding is therefore noteworthy but must be interpreted with utmost caution.

This study represents an initial, systematic step in bringing forth ORX antagonism to the clinic. The ultimate goal was to demonstrate target engagement and inform decisions to advance this line of work. The findings are a clear ‘Go’ signal to push forward in several important directions. First, additional laboratory studies are needed to determine whether SUV (or other ORX antagonists) selectively decreases reactivity to U-threat within clinical populations. Our data indicate SUV modulates anticipatory anxiety in relatively healthy adults. Given the multifaceted differences between healthy adults and patients, it remains to be tested whether the present findings will generalize, as well as the magnitude of SUV effects in high anxiety patients with or without a history of heavy alcohol use. Second, it is presently unclear whether SUV, and other dual orexin receptor antagonists, will be the ideal orexin-based therapies for psychiatric disorders. SUV is a readily available probe of the ORX system that was leveraged in the present study for systematic human investigation. It is important to note that the literature on functional differences between ORX1R and ORX2R is somewhat mixed. There is evidence that ORX1R plays a more prominent role in arousal, anxiety-like behavior, and alcohol use compared with ORX2R [57, 58]. A few studies have specifically shown that ORX1R inhibition blocks the anxiolytic-like effects of acute alcohol exposure [59]. Accordingly, there is widespread enthusiasm for the development of selective ORX1R receptors for human translation. Some evidence has also pointed to the role of ORX2R in alcohol-seeking behavior [60] and alcohol reinforcement [61]. Thus, additional human research is needed to better understand whether ORX1R, ORX2R, or dual receptor antagonism is most effective, and for whom.

The current study had numerous strengths including the within-subjects placebo-controlled design and the use of a well-validated laboratory paradigm. There were also several important limitations. We administered a single dose of SUV (10 mg) and examined startle reactivity during peak drug concentration. The dose-dependent effects and time course of SUV on startle reactivity to U-threat are therefore unclear. Future studies are needed to identify optimal dosing and to what extent SUV’s impact on startle is maintained over time. The study was open-label and there was no active control. Related, capsule blinding was only partially successful based on participant self-report. The study also lacked additional anxiety-related biomarkers and a fine-grained assessment of drug mechanisms. Lastly, the sample size was small, by intention, given the Fast-Fail, proof-of-concept design. The effect of SUV on startle reactivity to U-threat was robust; though it is possible the study was underpowered to detect drug-related differences in the other task conditions.

The current study is the first to demonstrate that a single dose of SUV dampens objective measures of anticipatory anxiety in humans. SUV had no impact on baseline (No-threat) startle nor startle reactivity to threats that are predictable (P-threat). ORX antagonism, therefore, targets and modifies our laboratory measure and may be a promising strategy for alleviating chronic arousal and disrupting stress-related alcohol use. The current findings serve as a preliminary ‘Go’ signal to pursue ORX antagonism as a potential novel pharmacological intervention for anxiety and addiction.

## References

[1] Barlow DH (2000). Unraveling the mysteries of anxiety and its disorders from the perspective of emotion theory. Am Psychol.

[2] Davis M, Walker DL, Miles L, Grillon C (2010). Phasic vs. sustained fear in rats and humans: Role of the extended amygdala in fear vs anxiety. Neuropsychopharmacology.

[3] Grillon C, Baas JM, Pine DS, Lissek S, Lawley M, Ellis V (2006). The benzodiazepine alprazolam dissociates contextual fear from cued fear in humans as assessed by fear-potentiated startle. Biol Psychiatry.

[4] Alvarez RP, Chen G, Bodurka J, Kaplan R, Grillon C (2011). Phasic and sustained fear in humans elicits distinct patterns of brain activity. Neuroimage.

[5] Davis M (2006). Neural systems involved in fear and anxiety measured with fear-potentiated startle. Am Psychol.

[6] Gorka SM, Lieberman L, Shankman SA, Phan KL (2017). Startle potentiation to uncertain threat as a psychophysiological indicator of fear-based psychopathology: an examination across multiple internalizing disorders. J Abnorm Psychol.

[7] Gorka SM, Kreutzer KA, Petrey KM, Radoman M, Phan KL (2020). Behavioral and neural sensitivity to uncertain threat in individuals with alcohol use disorder: associations with drinking behaviors and motives. Addict Biol.

[8] Schmitz A, Grillon C (2012). Assessing fear and anxiety in humans using the threat of predictable and unpredictable aversive events (the NPU-threat test). Nat Protoc.

[9] Grillon C, Baas J (2003). A review of the modulation of the startle reflex by affective states and its application in psychiatry. Clin Neurophysiol.

[10] Lang PJ, Bradley MM, Cuthbert BN (1990). Emotion, attention, and the startle reflex. Psychol Rev.

[11] Shankman SA, Nelson BD, Sarapas C, Robison-Andrew EJ, Campbell ML, Altman SE (2013). A psychophysiological investigation of threat and reward sensitivity in individuals with panic disorder and/or major depressive disorder. J Abnorm Psychol.

[12] Moberg CA, Curtin JJ (2009). Alcohol selectively reduces anxiety but not fear: startle response during unpredictable versus predictable threat. J Abnorm Psychol.

[13] Hefner KR, Curtin JJ (2012). Alcohol stress response dampening: selective reduction of anxiety in the face of uncertain threat. J Psychopharmacol.

[14] Kaye JT, Bradford DE, Curtin JJ (2006). Psychometric properties of startle and corrugator response in NPU, affective picture viewing, and resting state tasks. Psychophysiol.

[15] Gorka SM, Lieberman L, Shankman SA, Phan KL (2017). Association between neural reactivity and startle reactivity to uncertain threat in two independent samples. Psychophysiol.

[16] Gorka SM, Phan KL. Orexin modulation of stress reactivity as a novel targeted treatment for anxiety and alcohol use disorder. Neuropsychopharmacol. 2021;47:397–8. 10.1038/s41386-021-01120-4 PMC8616906 34341494

[17] Grillon C, Ernst M (2020). A way forward for anxiolytic drug development: testing candidate anxiolytics with anxiety-potentiated startle in healthy humans. Neurosci Biobehav Rev.

[18] Gimeno C, Dorado ML, Roncero C, Szerman N, Vega P, Balanza-Martinez V (2017). Treatment of comorbid alcohol dependence and anxiety disorder: review of the scientific evidence and recommendations for treatment. Front Psychiatry.

[19] Sakurai T, Amemiya A, Ishii M, Matsuzaki I, Chemelli RM, Tanaka H (1998). Orexins and orexin receptors: a family of hypothalamic neuropeptides and G protein-coupled receptors that regulate feeding behavior. Cell.

[20] de Lecea L, Kilduff TS, Peyron C, Gao X, Foye PE, Danielson PE (1998). The hypocretins: hypothalamus-specific peptides with neuroexcitatory activity. Proc Natl Acad Sci USA.

[21] Mahler SV, Moorman DE, Smith RJ, James MH, Aston-Jones G (2014). Motivational activation: a unifying hypothesis of orexin/hypocretin function. Nat Neurosci.

[22] Tsunematsu T, Yamanaka A (2012). The role of orexin/hypocretin in the central nervous system and peripheral tissues. Vitam Horm.

[23] Sakurai T, Nagata R, Yamanaka A, Kawamura H, Tsujino N, Muraki Y (2005). Input of orexin/hypocretin neurons revealed by a genetically encoded tracer in mice [published correction appears in Neuron. 2005 Jun 2;46(5):837]. Neuron.

[24] Sargin D (2019). The role of the orexin system in stress response. Neuropharmacology.

[25] Berridge CW, España RA, Vittoz NM (2010). Hypocretin/orexin in arousal and stress. Brain Res.

[26] Bonaventure P, Dugovic C, Shireman B, Preville C, Yun S, Lord B (2017). Evaluation of JNJ-54717793 a novel brain penetrant selective orexin 1 receptor antagonist in two rat models of panic attack provocation. Front Pharm.

[27] Chen X, Wang H, Lin Z, Li S, Li Y, Bergen HT (2014). Orexins (hypocretins) contribute to fear and avoidance in rats exposed to a single episode of footshocks. Brain Struct Funct.

[28] Johnson PL, Truitt W, Fitz SD, Minick PE, Dietrich A, Sanghani S (2010). A key role for orexin in panic anxiety. Nat Med.

[29] Akça ÖF, Uzun N, Kılınç İ (2020). Orexin A in adolescents with anxiety disorders. Int J Psychiatry Clin Pr.

[30] Gottschalk MG, Richter J, Ziegler C, Schiele MA, Mann J, Geiger MJ (2019). Orexin in the anxiety spectrum: association of a HCRTR1 polymorphism with panic disorder/agoraphobia, CBT treatment response and fear-related intermediate phenotypes. Transl Psychiatry.

[31] Moorman DE (2018). The hypocretin/orexin system as a target for excessive motivation in alcohol use disorders. Psychopharmacol (Berl).

[32] Hopf FW (2020). Recent perspectives on orexin/hypocretin promotion of addiction-related behaviors. Neuropharmacology.

[33] Lawrence AJ, Cowen MS, Yang HJ, Chen F, Oldfield B (2006). The orexin system regulates alcohol-seeking in rats. Br J Pharm.

[34] Voorhees CM, Cunningham CL (2011). Involvement of the orexin/hypocretin system in ethanol conditioned place preference. Psychopharmacol (Berl).

[35] Carvajal F, Alcaraz-Iborra M, Lerma-Cabrera JM, Valor LM, de la Fuente L, del Carmen Sanchez-Amate M (2015). Orexin receptor 1 signaling contributes to ethanol binge-like drinking: pharmacological and molecular evidence. Behav Brain Res.

[36] Olney JJ, Navarro M, Thiele TE (2015). Binge-like consumption of ethanol and other salient reinforcers is blocked by orexin-1 receptor inhibition and leads to a reduction of hypothalamic orexin immunoreactivity. Alcohol Clin Exp Res.

[37] Anderson RI, Becker HC, Adams BL, Jesudason CD, Rorick-Kehn LM (2014). Orexin-1 and orexin-2 receptor antagonists reduce ethanol self-administration in high-drinking rodent models. Front Neurosci.

[38] Moorman DE, Aston-Jones G (2009). Orexin-1 receptor antagonism decreases ethanol consumption and preference selectively in high-ethanol—preferring Sprague—Dawley rats. Alcohol.

[39] Richards JK, Simms JA, Steensland P, Taha SA, Borgland SL, Bonci A (2008). Inhibition of orexin-1/hypocretin-1 receptors inhibits yohimbine-induced reinstatement of ethanol and sucrose seeking in Long-Evans rats. Psychopharmacol (Berl).

[40] Bayerlein K, Kraus T, Leinonen I, Pilniok D, Rotter A, Hofner B (2011). Orexin A expression and promoter methylation in patients with alcohol dependence comparing acute and protracted withdrawal. Alcohol.

[41] Yee KL (2018). Safety, tolerability, and pharmacokinetics of suvorexant: a randomized rising-dose trial in healthy men. Clin Drug Investig.

[42] Folstein MF, Luria R (1973). Reliability, validity, and clinical application of the Visual Analogue Mood Scale. Psychol Med.

[43] Johanson CE, Uhlenhuth EH (1980). Drug preference and mood in humans: d-amphetamine. Psychopharmacol (Berl).

[44] McNair DM, Lorr M, Droppleman LF. Manual Profile of Mood States. *Educational and Industrial Testing Service*. 1971;756.

[45] Radoman M, Crane NA, Gorka SM, Weafer J, Langenecker SA, de Wit H (2021). Striatal activation to monetary reward is associated with alcohol reward sensitivity. Neuropsychopharmacology.

[46] Lovibond PF, Lovibond SH (1995). The structure of negative emotional states: comparison of the Depression Anxiety Stress Scales (DASS) with the beck depression and anxiety inventories. Behav Res Ther.

[47] Babor TF, de la Fuente JR, Saunders J, Grant M. AUDIT: the alcohol use disorders identification test: guidelines for use in primary health care. *Geneva: World Health Organization*. 1992.

[48] Gorka SM, Nelson BD, Shankman SA (2013). Startle response to unpredictable threat in comorbid panic disorder and alcohol dependence. Drug Alcohol Depend.

[49] Gorka SM, Lieberman L, Phan KL, Shankman SA (2016). Association between problematic alcohol use and reactivity to uncertain threat in two independent samples. Drug Alcohol Depend.

[50] Blumenthal TD, Cuthbert BN, Filion DL, Hackley S, Lipp OV, van Boxtel A (2005). Committee report: guidelines for human startle eyeblink electromyographic studies. Psychophysiology.

[51] Johnson PL, Samuels BC, Fitz SD, Lightman SL, Lowry CA, Shekhar A (2012). Activation of the orexin 1 receptor is a critical component of CO2-mediated anxiety and hypertension but not bradycardia. Neuropsychopharmacology.

[52] Walker LC, Lawrence AJ (2017). The role of orexins/hypocretins in alcohol use and abuse. Curr Top Behav Neurosci.

[53] Caldirola D, Alciati A, Cuniberti F, Perna G (2021). Experimental drugs for panic disorder: an updated systematic review. J Exp Pharm.

[54] Han Y, Yuan K, Zheng Y, Lu L (2020). Orexin receptor antagonists as emerging treatments for psychiatric disorders. Neurosci Bull.

[55] Russell JL, Spiller HA (2019). Retrospective assessment of toxicity following exposure to Orexin pathway modulators modafinil and suvorexant. Toxicol Commun.

[56] Ito H, Ogawa Y, Shimojo N, Kawano S (2021). Suvorexant poisoning in a patient with cirrhosis and renal failure. Cureus.

[57] Lopez MF, Moorman DE, Aston-Jones G, Becker HC (2016). The highly selective orexin/hypocretin 1 receptor antagonist GSK1059865 potently reduces ethanol drinking in ethanol dependent mice. Brain Res.

[58] Johnson PL, Molosh A, Fitz SD, Truitt WA, Shekhar A (2012). Orexin, stress, and anxiety/panic states. Prog Brain Res.

[59] Morales-Mulia M (2019). Intra-accumbal orexin-1 receptor inhibition prevents the anxiolytic-like effect of ethanol and leads to increases in orexin-A content and receptor expression. Pharm Biochem Behav.

[60] Kastman HE, Blasiak A, Walker L, Siwiec M, Krstew EV, Gundlach AL (2016). Nucleus incertus Orexin2 receptors mediate alcohol seeking in rats. Neuropharmacology.

[61] Shoblock JR, Welty N, Aluisio L, Fraser I, Motley ST, Morton K (2011). Selective blockade of the orexin-2 receptor attenuates ethanol self-administration, place preference, and reinstatement. Psychopharmacol (Berl).

